# Supplementary material for: Glycemic control during TB treatment among Filipinos: The Starting Anti-Tuberculosis Treatment Cohort Study
Source: PLOS Glob Public Health. 2024 May 2;4(5):e0003156. doi: 10.1371/journal.pgph.0003156 (PMC11065219; doi:10.1371/journal.pgph.0003156)
Supplement: S2 Checklist — (DOC) [file pgph.0003156.s002.doc]

STROBE Statement—Checklist of items that should be included in reports of ***cohort studies***

|  | Item No | Recommendation |  | Response |
| --- | --- | --- | --- | --- |
| **Title and abstract** | 1 | (*a*) Indicate the study’s design with a commonly used term in the title or the abstract | Yes | Study design is indicated in the abstract: “This prospective cohort study aimed to investigate glycemic control longitudinally among Filipino adults undergoing TB treatment using mixed-effects linear and logistic regression” |
|  |  | (*b*) Provide in the abstract an informative and balanced summary of what was done and what was found | Yes | Study objective, methods and results are stated in the abstract. |
| Introduction | | |  |  |
| Background/rationale | 2 | Explain the scientific background and rationale for the investigation being reported | Yes | Rationale and relevant literature are stated in the introduction section. |
| Objectives | 3 | State specific objectives, including any prespecified hypotheses | Yes | A statement at the end of the introduction specifies the specific goals and objectives. “The aim of this study was to investigate glycemic control among DM patients receiving Directly Observed Treatment for the Treatment of TB (TB-DOTS) in the Philippines and the factors influencing glycemic control, in order to generate evidence to inform and improve targeted support for DM management in the context of limited resources” |
| Methods | | |  |  |
| Study design | 4 | Present key elements of study design early in the paper | Yes | Study design is stated in the first subsection of Methods. “This study was part of the Starting Anti-TB Treatment (St-ATT) cohort, a prospective cohort study of people starting treatment for drug-sensitive and drug-resistant TB in public TB-DOTS clinics in the Philippines (ISRCTN16347615” |
| Setting | 5 | Describe the setting, locations, and relevant dates, including periods of recruitment, exposure, follow-up, and data collection | Yes | Setting, contexts, dates of inclusion, are fully described in the method section under “Setting”, “Participant”, and “data collection” headings. |
| Participants | 6 | (*a*) Give the eligibility criteria, and the sources and methods of selection of participants. Describe methods of follow-up | Yes | Study population is described is the method section (Study population headline), as well as selection criteria.  “Non-pregnant adults (≥18 years) who were enrolled at participating sites, with either a previous diagnosis of DM or a new diagnosis (Table 1), and with at least one HbA1c measurement collected. Participants with transient hyperglycemia (Table 1) were excluded from the analysis. Informed consent procedures were performed by St-ATT research nurses. Written informed consent was obtained from all participants prior to participation in the local language (﻿Filipino, Cebuano, Hilagaynon) or English” |
|  |  | (*b*) For matched studies, give matching criteria and number of exposed and unexposed | N/A | Not applicable |
| Variables | 7 | Clearly define all outcomes, exposures, predictors, potential confounders, and effect modifiers. Give diagnostic criteria, if applicable | Yes | Standardized variable definitions were used, which are presented in the “Outcomes” and “Exposures” subsections of the method section. Validity of all variables was previously assessed for misunderstanding and potential cofounders. |
| Data sources/ measurement | 8* | For each variable of interest, give sources of data and details of methods of assessment (measurement). Describe comparability of assessment methods if there is more than one group | Yes | Data collection and measurement is described in the methods section, under the ‘Data Collection”, “Outcomes” and “Measurements” sections. |
| Bias | 9 | Describe any efforts to address potential sources of bias | Yes | We notably tried to reduce bias by excluding patients who likely had transient hyperglycaemia caused by acute TB infection, rather than true diabetes. This is described in the “Participants” section of the Methods. |
| Study size | 10 | Explain how the study size was arrived at | Yes | This is described in the methods section.  “This study was part of the Starting Anti-TB Treatment (St-ATT) cohort, a prospective cohort study of 902 people starting treatment for drug-sensitive and drug-resistant TB in public TB-DOTS clinics in the Philippines”. Study size was dependent on yield of DM screening performed at enrolment to TB treatment and tri-monthly throughout the duration of TB treatment. |
| Quantitative variables | 11 | Explain how quantitative variables were handled in the analyses. If applicable, describe which groupings were chosen and why | Yes | Definitions of all categories for variables are presented in Table 2 and Table 3. |
| Statistical methods | 12 | (*a*) Describe all statistical methods, including those used to control for confounding | Yes | These are described in the method section.  Univariable associations with HbA1c at any point in time were investigated using mixed-effects linear regression with a random intercept for individuals and a random slope for time (days from the start of treatment). Visual inspection showed that HbA1c % decreased on average as treatment progressed, but the rate of decrease was non-linear. Therefore, time was transformed using a square root function with a constant of 0.01 added to achieve positivity of values (54). Differential changes in HbA1c over time within strata of categorical exposures were investigated by fitting an interaction between time and each exposure. Restricted maximum likelihood (REML) was the likelihood estimator, being more appropriate for smaller samples. Likelihood Ratio Test (LRT) of fixed effects cannot be used with models fit using REML (55); a global Wald test with a small-sample adjustment for fixed effects (56) (P<0.1) was used to assess associations. Between-site variation (clustering) was investigated by comparing models with and without a random intercept for site (LRT P-value <0.01).  A multivariable model was developed using forward step-wise selection of variables in four blocks, starting with socio-demographic characteristics, followed by anthropometric variables, then DM-related variables, and TB-related variables. Central obesity was included in the final model a priori. Variables in each block were tested for inclusion in the order of strength of effect in univariable analysis. Selection of a final model was based on inclusion of factors associated with the outcome based on a Wald test P value of <0.1 in univariable analysis and retained if meeting this criterion after adjustment for other covariates. After adjustment for all factors remaining in the multivariable model, interactions terms between time and each covariate, and between covariates were tested. **Analysis of predictors of poor glycemic control**  Associations between participant characteristics and poor glycemic control were investigated using logistic and multinomial regression for the binary and categorical outcomes (Table 2), respectively. The same multivariable model-building approach described above was also used here. Logistic regression was used to investigate associations with controlled versus uncontrolled glycemia. Multinomial regression was used to investigate associations with the degree of poor glycemic control (i.e., controlled versus initially-uncontrolled and consistently-uncontrolled glycemia). All data were analyzed with Stata (Version 15, College Station, Texas: StataCorp LP). |
|  |  | (*b*) Describe any methods used to examine subgroups and interactions | Yes | This is described in the method section. Interaction terms were systematically examined. |
|  |  | (*c*) Explain how missing data were addressed | Yes | Missing data were not imputed. We described missingness of the main outcome variable, HbA1c, in Table 3, and the implications of missing HbA1c and other data are addressed in the discussion section under “Study strengths, limitations and further research”. Mixed-effects regression models take a powerful and flexible approach to handling missing data, which is why it was used as part of our analysis.  In the discussion, we also address implications of missing data for key variables:  “There was not complete or precise enough data available on the duration of DM disease prior to enrollment to include in the analysis. Persons reporting a previous DM diagnosis self-reported the date of DM diagnosis; more than half of participants did not respond. Duration of DM disease could be an unmeasured predictor of hyperglycemia. Future research of persons with DM within TB cohorts could benefit from verifying outside DM diagnosis using medical record review.  Glucose-lowing medication exposure over time is an important variable which was not measured, but likely an important time-dependent exposure. Measuring medication exposure over time using electronic monitoring of adherence and prescription data about medication formulation and dosage, or direct measurement of medication concentration in dried blood samples could provide useful data.  Exclusions of participants with fewer HbA1c results could lead to bias in the analysis of associations with uncontrolled glycemia, causing over- or underestimation of the strength and association between patterns of glycemic control and exposures, or failure to detect associations during the model-building process. Furthermore, participants in Manila had fewer HbA1c results than those in other regions. To minimize the exclusion of patients with insufficient HbA1c results, research nurses conducted targeted follow-ups for individuals with fewer than two results, making efforts to mitigate potential bias.” |
|  |  | (*d*) If applicable, explain how loss to follow-up was addressed | N/A | Non applicable |
|  |  | (*e*) Describe any sensitivity analyses | N/A | Non applicable |
| Results | | |  |  |
| Participants | 13* | (a) Report numbers of individuals at each stage of study—eg numbers potentially eligible, examined for eligibility, confirmed eligible, included in the study, completing follow-up, and analysed | Yes | This is included as Figure 1. We describe the number of participants included in each analyses in Tables 4 and 5, and in S2 and S3 Figures. |
|  |  | (b) Give reasons for non-participation at each stage | Yes | This is described at the beginning of result section under “Participants”.  “Of 901 participants enrolled in the St-ATT cohort (47), 200 had previously diagnosed or newly diagnosed DM per St-ATT case definitions (Table 1). One person previously-diagnosed with DM with no HbA1c measurements was excluded. A further 48 suspected DM cases were identified as a result of external reported diagnoses or HbA1c results during follow-up. Fifty-nine suspected DM cases, mostly diagnosed at the time of enrollment, were transiently hyperglycemic (Table 1) and excluded from analyses.” |
|  |  | (c) Consider use of a flow diagram | Yes | This is included as Figure 1. |
| Descriptive data | 14* | (a) Give characteristics of study participants (eg demographic, clinical, social) and information on exposures and potential confounders | Yes | Table 3 describes the participants included.  [Table 3. Socio-demographic, anthropometric, tuberculosis- and diabetes mellitus-related characteristics of 188 Starting Anti-TB Treatment Cohort Study participants with a diabetes mellitus (DM) comorbidity by region.] |
|  |  | (b) Indicate number of participants with missing data for each variable of interest | Yes | The total numbers of recorded data for each variable are stated in variable row of each table |
|  |  | (c) Summarise follow-up time (eg, average and total amount) | Yes | Typical duration of treatment described under “Data collection”: Participants were followed at monthly appointments until treatment exit (typically 6-12 months for drug-sensitive TB [DS-TB]).  Actual time in treatment approximated by number of HbA1c readings, captured at baseline and tri-monthly, were available. This is described in results “More than half of the sample (N=97) had data for baseline, three- and six-months (Table 3, S1 Fig),” and in Table 3. |
| Outcome data | 15* | Report numbers of outcome events or summary measures over time | Yes | All numbers are reported in Tables. |
| Main results | 16 | (*a*) Give unadjusted estimates and, if applicable, confounder-adjusted estimates and their precision (eg, 95% confidence interval). Make clear which confounders were adjusted for and why they were included | Yes | Unadjusted estimates and their 95% confidence intervals are provided in Supplementary Tables 2 and 3. |
|  |  | (*b*) Report category boundaries when continuous variables were categorized | Yes | Category boundaries are displayed in variable headings in the tables, where applicable (e.g., age groups, glycaemic control groupings) |
|  |  | (*c*) If relevant, consider translating estimates of relative risk into absolute risk for a meaningful time period | No |  |
| Other analyses | 17 | Report other analyses done—eg analyses of subgroups and interactions, and sensitivity analyses | Yes | We described interaction terms in methods, results, and depicted interactions graphically in Supplementary Figure 2 and 3. |
| Discussion | | |  |  |
| Key results | 18 | Summarise key results with reference to study objectives | Yes | Key results are described at the beginning of discussion section (page 23). They also are summarized in the conclusion |
| Limitations | 19 | Discuss limitations of the study, taking into account sources of potential bias or imprecision. Discuss both direction and magnitude of any potential bias | Yes | Description of limitations is done under “Study strengths, limitations and further research” heading in the discussion (page 25). |
| Interpretation | 20 | Give a cautious overall interpretation of results considering objectives, limitations, multiplicity of analyses, results from similar studies, and other relevant evidence | Yes | References were included as much as possible, and discussed. Limitations were addressed in the discussion |
| Generalisability | 21 | Discuss the generalisability (external validity) of the study results | Yes | This is addressed in the Discussion sections: “Findings from this work may be generalizable to countries with high TB-DM burden striving to achieve Universal Health (UHC) schemes against a background of scare resources and weak health systems, especially in Southeast Asia. However, in this study, participants commonly accessed free glucose-lowering medications through public services; findings may not be generalizable to settings where diabetes is not addressed under a UHC scheme.” |
| Other information | | |  |  |
| Funding | 22 | Give the source of funding and the role of the funders for the present study and, if applicable, for the original study on which the present article is based |  | Funding information were displayed upon submission but not included in the manuscript, as requested. |

*Give information separately for exposed and unexposed groups.

**Note:** An Explanation and Elaboration article discusses each checklist item and gives methodological background and published examples of transparent reporting. The STROBE checklist is best used in conjunction with this article (freely available on the Web sites of PLoS Medicine at http://www.plosmedicine.org/, Annals of Internal Medicine at http://www.annals.org/, and Epidemiology at http://www.epidem.com/). Information on the STROBE Initiative is available at http://www.strobe-statement.org.
