# Supplementary material for: Glycemic control during TB treatment among Filipinos: The Starting Anti-Tuberculosis Treatment Cohort Study
Source: PLOS Glob Public Health. 2024 May 2;4(5):e0003156. doi: 10.1371/journal.pgph.0003156 (PMC11065219; doi:10.1371/journal.pgph.0003156)
Supplement: S1 Fig — Distribution of individual glycosylated hemoglobin results at pre-specified measurement time points during TB treatment among 188 Patients with a with a newly-diagnosed (A) or previously-diagnosed (B) DM Comorbidity. (DOCX) [file pgph.0003156.s003.docx]

**S1 Figure.** Distribution of individual glycosylated hemoglobin results at pre-specified measurement time points during TB treatment among 188 Patients with a with a newly-diagnosed (A) or previously-diagnosed (B) DM Comorbidity.


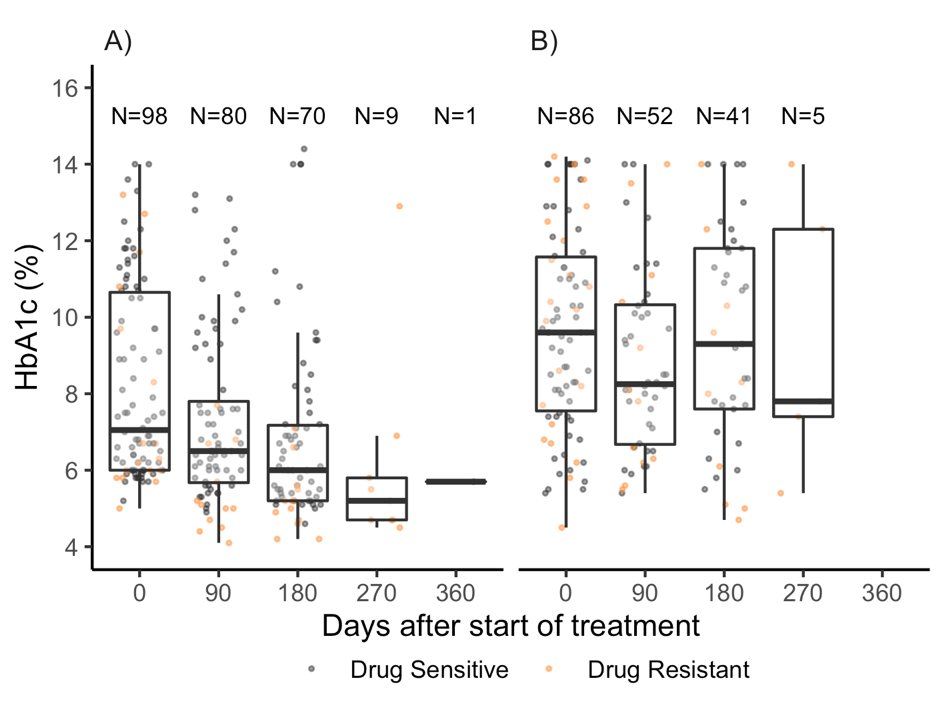


Legend: diabetes mellitus, DM; glycosylated hemoglobin, HbA1c; interquartile range, IQR, tuberculosis, TB; Directly Observed Treatment for the Treatment of Tuberculosis, TB-DOTS. Footnote: Boxes indicate median and IQR. Measurements were taken at time of enrollment and within +/- 14 days of scheduled follow-up at day 90, 180, 270, 360.
