# Supplementary material for: Glycemic control during TB treatment among Filipinos: The Starting Anti-Tuberculosis Treatment Cohort Study
Source: PLOS Glob Public Health. 2024 May 2;4(5):e0003156. doi: 10.1371/journal.pgph.0003156 (PMC11065219; doi:10.1371/journal.pgph.0003156)
Supplement: S2 Fig — Predicted mean glycosylated hemoglobin (HbA1c, %) at any time, with corresponding 95% confidence interval with a newly-diagnosed (A) or previously-diagnosed (B) diabetes mellitus comorbidity, overall and stratified by central obesity by mixed-effects linear regression analysis. (DOCX) [file pgph.0003156.s004.docx]

**S2 Figure.** Predicted mean glycosylated hemoglobin (HbA1c, %) at any time, with corresponding 95% confidence interval with a newly-diagnosed (A) or previously-diagnosed (B) diabetes mellitus comorbidity, overall and stratified by central obesity by mixed-effects linear regression analysis.

**
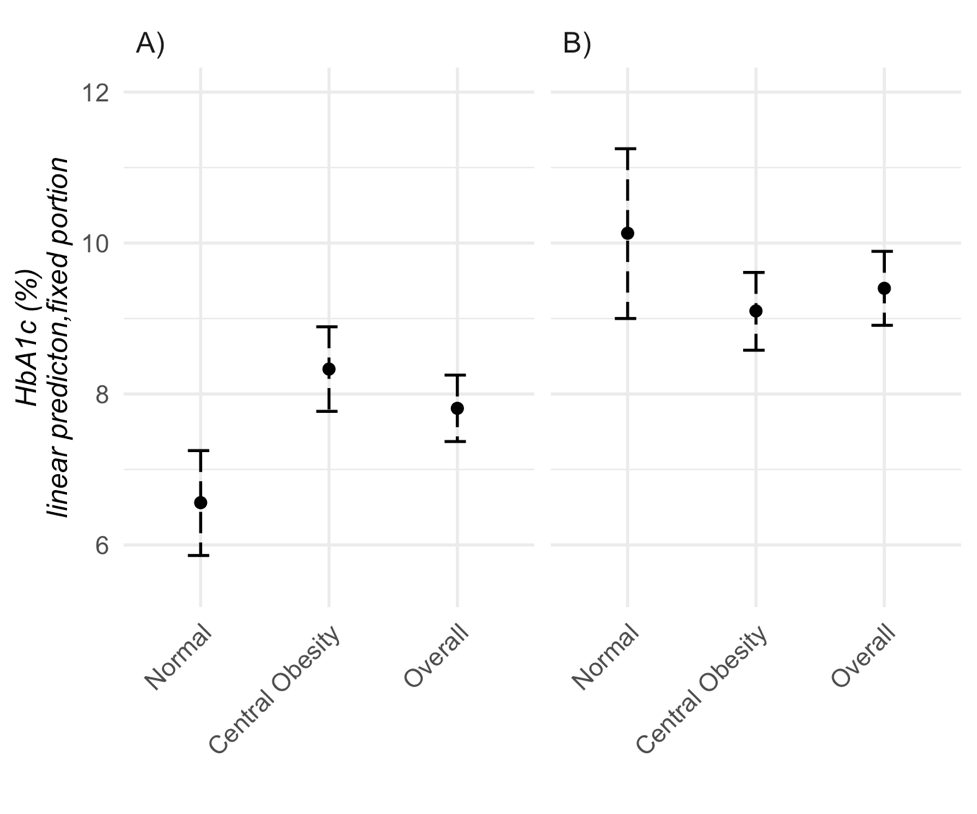
**

Legend: DM, diabetes mellitus; HbA1c, glycosylated hemoglobin; TB-DOTS, Directly Observed Treatment for the Treatment of Tuberculosis. Footnotes: Results generated from final multivariable model, which adjusted for adjusted for central obesity at baseline, blood pressure at baseline, timing of DM diagnosis, and interaction terms between central obesity and timing of DM diagnosis, and between time and TB treatment regimen. Model accounted for a random slope for individuals. There were not significant differences in mean HbA1c at baseline using a one-way analysis of variance (ANOVA) test between those with drug-sensitive versus drug-resistant TB.
