# Supplementary material for: Glycemic control during TB treatment among Filipinos: The Starting Anti-Tuberculosis Treatment Cohort Study
Source: PLOS Glob Public Health. 2024 May 2;4(5):e0003156. doi: 10.1371/journal.pgph.0003156 (PMC11065219; doi:10.1371/journal.pgph.0003156)
Supplement: S3 Fig — (DOCX) [file pgph.0003156.s005.docx]

**S3 Figure. Predicted mean change in glycosylated hemoglobin (HbA1c, %) with corresponding 95% confidence interval by drug resistance status by mixed-effects linear regression analysis.**


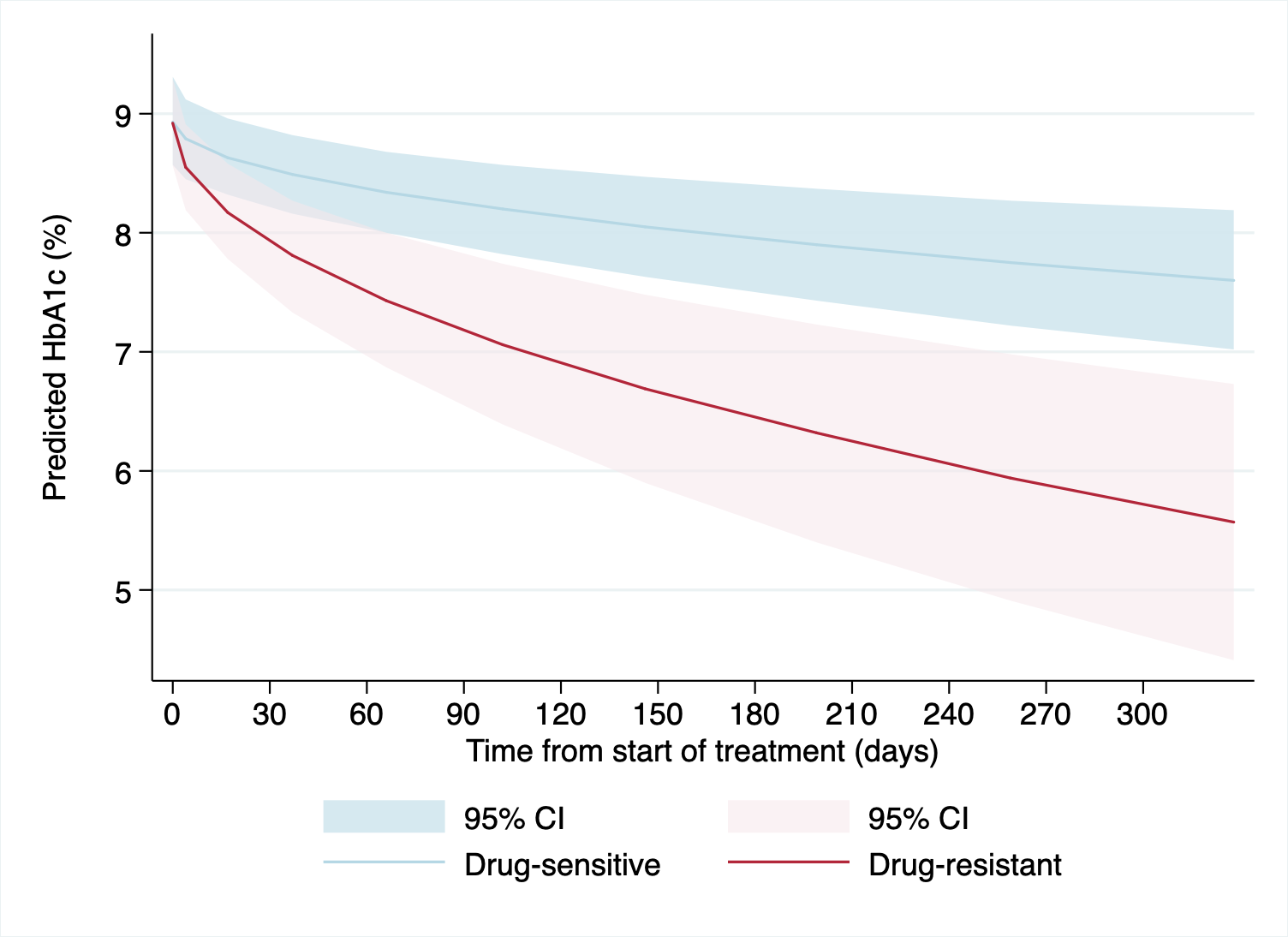


Legend: CI, confidence interval; HbA1c, glycosylated hemoglobin

Footnotes: Results generated from final multivariable model, which adjusted for adjusted for central obesity at baseline, blood pressure at baseline, timing of diabetes mellitus (DM) diagnosis, and interaction terms between central obesity and timing of DM diagnosis, and between time and tuberculosis treatment regimen. Model accounted for a random slope for individuals. There were not significant differences in mean HbA1c at baseline using a one-way analysis of variance (ANOVA) test between those with drug-sensitive versus drug-resistant TB.
