# Supplementary material for: Glycemic control during TB treatment among Filipinos: The Starting Anti-Tuberculosis Treatment Cohort Study
Source: PLOS Glob Public Health. 2024 May 2;4(5):e0003156. doi: 10.1371/journal.pgph.0003156 (PMC11065219; doi:10.1371/journal.pgph.0003156)
Supplement: S1 Table — Abbreviations: BMI, Body Mass Index; DM, diabetes mellitus; HT, cartridge-based nucleic acid amplification test (Cephid), GeneXpert; hypertension; Standard deviation (SD); DSSM, Direct sputum smear microscopy (DSSM); Philippine peso (PHP), TB, Tuberculosis (TB), Hypertension (HT). a Amongst those with > 2 glycosylated hemoglobin (HbA1c) results: Uncontrolled (at least two study-measured HbA1c results equal to or greater than 8%); controlled (at least two study-measured HbA1c results less than 8%). Binary glycemic control outcome is not mutually exclusive to degree of glycemic control outcome. b Amongst those with > 3 HbA1c results: Controlled (all HbA1c values were less than 8%); initially-uncontrolled (baseline HbA1c measurement was greater than 8%, and all subsequent measurements were less than 8%); consistently-uncontrolled (all HbA1c values were equal to or greater than 8%). Degree of glycemic control outcome is not mutually exclusive with binary glycemic control outcome. c Any of the following plans: Philippines Health Insurance plan, Social Security, or Government Service Insurance. d Based on waist-to-hip ratio >0.85 for women and >0.9 for men used by the World Health Organization for use in diagnostic criteria for metabolic syndrome [49]. e Normal (Systolic blood pressure (SBP) <120 and diastolic blood pressure (DBP) <80 mm Hg); elevated (SBP 120–129 mm Hg and DBP <80 mm Hg); Stage 1 Hypertension (SBP 130–139 mm Hg and DBP 80–89 mm Hg); and Stage 2 hypertension (SBP > 140 mm Hg and DBP > 90 mm Hg), by the 2017 American College of Cardiology and American Heart Association guidelines [50]. f BMI according to WHO criteria for adults: underweight (BMI<18.5 kg/m2), normal (BMI 18.5–25.0), overweight (25.0–29.9), obese (BMI >30) [48]. g Confirmed by GeneXpert (Cepheid), a cartridge-based nucleic acid amplification test for simultaneous rapid tuberculosis diagnosis and rapid antibiotic sensitivity test, or by direct sputum smear microscopy. h Self-reported at enrol [file pgph.0003156.s006.docx]

**S1 Table.** Socio-demographic, anthropometric, TB- and DM-related characteristics at enrollment into TB treatment of the 188 study participants, comparing to the subset of 151 participants included in the binary logistic regression

|  | Total  (N=188) | Controlled versus uncontrolled^a^ | | Degree of glycemic control^b^ | |
| --- | --- | --- | --- | --- | --- |
|  |  | Included in analysis (%)  (N=151) | Excluded from analysis (%)  (N=37) | Included in analysis (%)  (N=113) | Excluded from analysis (%)  (N=75) |
| Age, years |  |  |  |  |  |
| Mean, SD | 50.6, 12.6 | 50.3, 12.3 | 52.2, 14.0 | 50.3 (12.8) | 51.2, 12.4 |
| Median, Range | 51.0  18.0, 83.0 | 50.0  18.0, 77.0 | 52.0  22.0, 83.0 | 51.0  18.0, 77.0 | 51.0  22.0, 83.0 |
| Sex |  |  |  |  |  |
| Male | 131 (69.7) | 105 (69.5) | 26 (70.3) | 81 (71.7) | 50 (66.7) |
| Region |  |  |  |  |  |
| Negros Occidental | 77 (41.0) | 66 (43.7) | 11 (29.7) | 54 (47.8) | 23 (30.7) |
| Cebu | 83 (44.1) | 70 (46.4) | 13 (35.1) | 55 (48.7) | 28 (37.3) |
| Manila | 28 (14.9) | 15 (9.9) | 13 (35.1) | 4 (3.5) | 24 (32.0) |
| Residential area |  |  |  |  |  |
| Urban | 48 (25.5) | 30 (19.9) | 18 (48.6) | 17 (15.0) | 31 (41.3) |
| Peri-urban | 86 (45.7) | 76 (50.3) | 10 (27.0) | 61 (54.0) | 25 (33.3) |
| Rural | 54 (28.7) | 45 (29.8) | 9 (24.3) | 35 (31.0) | 19 (25.3) |
| Absolute annual household income level |  |  |  |  |  |
| Less than 5,000 PHP | 73 (39.0) | 63 (42.0) | 10 (27.0) | 48 (42.5) | 25 (33.8) |
| 5000, 9999 PHP | 50 (26.7) | 39 (26.0) | 11 (29.7) | 31 (27.4) | 19 (25.7) |
| >10,000 PHP | 64 (34.2) | 48 (32.0) | 16 (43.2) | 34 (30.1) | 30 (40.5) |
| Marital status |  |  |  |  |  |
| Single | 37 (19.7) | 29 (19.2) | 8 (21.6) | 20 (17.7) | 17 (22.7) |
| Married | 134 (71.3) | 109 (72.2) | 25 (67.6) | 82 (72.6) | 52 (69.3) |
| Divorced or separated | 3 (1.6) | 1 (0.7) | 2 (5.4) | 1 (0.9) | 2 (2.7) |
| Widowed | 14 (7.4) | 12 (7.9) | 2 (5.4) | 10 (8.8) | 4 (5.3) |
| Unemployed | 123 (65.4) | 100 (66.2) | 23 (62.2) | 78 (69.0) | 45 (60.0) |
| Higher than primary school education | 143 (76.5) | 115 (76.7) | 28 (75.7) | 87 (77.0) | 56 (75.7) |
| Possess health insurance^c^ | 114 (66.3) | 93 (67.4) | 21 (61.8) | 69 (66) | 46 (67) |
| Central obesity^d^ |  |  |  |  |  |
| Normal | 57 (30.3) | 49 (32.5) | 8 (21.6) | 39 (34.5) | 18 (24.0) |
| Central obesity | 131 (69.7) | 102 (67.5) | 29 (78.4) | 74 (65.5) | 57 (76.0) |
| Blood pressure^e^ |  |  |  |  |  |
| Normal | 73 (42.7) | 60 (44.8) | 13 (35.1) | 46 (45.1) | 27 (39.1) |
| Elevated | 16 (9.4) | 12 (9.0) | 4 (10.8) | 10 (9.8) | 6 (8.7) |
| Stage 1 Hypertension | 47 (27.5) | 43 (32.1) | 4 (10.8) | 33 (32.4) | 14 (20.3) |
| Stage 2 Hypertension | 35 (20.5) | 19 (14.2) | 16 (43.2) | 13 (12.7) | 22 (31.9) |
| BMI classification^f^ |  |  |  |  |  |
| Normal | 114 (61.0) | 90 (60.0) | 24 (64.9) | 61 (54.0) | 53 (71.6) |
| Underweight | 45 (24.1) | 36 (24.0) | 9 (24.3) | 32 (28.3) | 13 (17.6) |
| Overweight | 26 (13.9) | 22 (14.7) | 4 (10.8) | 18 (15.9) | 8 (10.8) |
| Obese | 2 (1.1) | 2 (1.3) | 0 | 2 (1.8) | 0 |
| Type of TB treatment facility |  |  |  |  |  |
| Public hospital | 51 (27.3) | 34 (22.7) | 17 (45.9) | 25 (22.1) | 26 (35.1) |
| City Health Center | 82 (43.9) | 71 (47.3) | 11 (29.7) | 53 (46.9) | 29 (39.2) |
| Rural Health Unit | 54 (28.9) | 45 (30.0) | 9 (24.3) | 35 (31.0) | 19 (25.7) |
| New versus relapse TB case |  |  |  |  |  |
| New | 119 (63.6) | 96 (64.0) | 23 (62.2) | 72 (63.7) | 47 (63.5) |
| Relapse | 68 (36.4) | 54 (36.0) | 14 (37.8) | 41 (36.3) | 27 (36.5) |
| Basis of TB diagnosis |  |  |  |  |  |
| Clinically diagnosed | 62 (33.2) | 51 (34.0) | 11 (29.7) | 39 (34.5) | 23 (31.1) |
| Bacteriologically-confirmed^g^ | 125 (66.8) | 99 (66.0) | 26 (70.3) | 74 (65.5) | 51 (68.9) |
| Duration of TB symptoms prior to treatment (weeks) |  |  |  |  |  |
| Mean, SD | 8.4, 8.5 | 8.4, 7.1 | 8.7, 12.9 | 8.4, 7.0 | 8.5, 10.5 |
| Median, Range | 6.0  0.0, 80.7 | 6.1  0.0, 40.4 | 6.0  0.0, 80.7 | 6.1  0.0, 40.4 | 5.9  0.0, 80.7 |
| TB regimen |  |  |  |  |  |
| Drug sensitive | 147 (78.2) | 118 (78.1) | 29 (78.4) | 87 (77.0) | 60 (80.0) |
| Drug resistant | 41 (21.8) | 33 (21.9) | 8 (21.6) | 26 (23.0) | 15 (20.0) |
| Timing of DM diagnosis |  |  |  |  |  |
| Newly diagnosed | 99 (52.7) | 90 (59.6) | 9 (24.3) | 71 (62.8) | 28 (37.3) |
| Previously diagnosed | 89 (47.3) | 61 (40.4) | 28 (75.7) | 42 (37.2) | 47 (62.7) |
| Report of glucose-lowering medications during TB treatment^g^ | 132 (70.2) | 102 (67.5) | 30 (81.1) | 72 (63.7) | 60 (80.0) |
| Report of any DM complication^i^ during TB treatment | 144 (76.6) | 111 (73.5) | 33 (89.2) | 86 (76.1) | 58 (77.3) |

| Abbreviations: BMI, Body Mass Index; DM, diabetes mellitus; HT, cartridge-based nucleic acid amplification test (Cephid), GeneXpert; hypertension; Standard deviation (SD); DSSM, Direct sputum smear microscopy (DSSM); Philippine peso (PHP), TB, Tuberculosis (TB), Hypertension (HT) | |
| --- | --- |
| a | Amongst those with > 2 glycosylated hemoglobin (HbA1c) results: Uncontrolled (at least two study-measured HbA1c results equal to or greater than 8%); controlled (at least two study-measured HbA1c results less than 8%). Binary glycemic control outcome is not mutually exclusive to degree of glycemic control outcome |
| b | Amongst those with > 3 HbA1c results: Controlled (all HbA1c values were less than 8%); initially-uncontrolled (baseline HbA1c measurement was greater than 8%, and all subsequent measurements were less than 8%); consistently-uncontrolled (all HbA1c values were equal to or greater than 8%). Degree of glycemic control outcome is not mutually exclusive with binary glycemic control outcome. |
| c | Any of the following plans: Philippines Health Insurance plan, Social Security, or Government Service Insurance |
| d | Based on waist-to-hip ratio >0.85 for women and >0.9 for men used by the World Health Organization for use in diagnostic criteria for metabolic syndrome (World Health Organization 2008). |
| e | Normal (Systolic blood pressure (SBP) <120 and diastolic blood pressure (DBP) <80 mm Hg); elevated (SBP 120-129 mm Hg and DBP <80 mm Hg); Stage 1 Hypertension (SBP 130-139 mm Hg and DBP 80-89 mm Hg); and Stage 2 hypertension (SBP > 140 mm Hg and DBP > 90 mm Hg), ﻿by the 2017 American College of Cardiology and American Heart Association guidelines (Whelton *et al.* 2018) |
| f | BMI according to WHO criteria for adults: underweight (BMI<18.5 kg/m^2)^, normal (BMI 18.5–25.0), overweight (25.0-29.9), obese (BMI >30) (World Health Organizaztion 2021). |
| g | Confirmed by GeneXpert (Cepheid), a cartridge-based nucleic acid amplification test for simultaneous rapid tuberculosis diagnosis and rapid antibiotic sensitivity test, or by direct sputum smear microscopy. |
| h | Self-reported at enrollment or point during TB treatment |
|  |  |
| i | After enrollment in TB treatment, report of experiencing any of the Concurrent Tuberculosis and Diabetes Mellitus Consortium (TANDEM) study DM complications (Ugarte-Gil *et al.* 2020): ever lost a limb or digit not through trauma, ever had a bypass or stenting surgery in limbs, non-healing wound for three or more months, heart attack, stroke, bypass or stenting heart surgery, diagnosis of angina or heart failure, cataract or laser eye surgery, glaucoma, acquired blindness not due to trauma, difficulty seeing or disturbed vision, renal failure. Additionally, the measure captures if participant had any symptom of distal symmetrical peripheral neuropathy using the Michigan Neuropathy Screening Instrument (Feldman *et al.* 1994). |
