# Supplementary material for: Glycemic control during TB treatment among Filipinos: The Starting Anti-Tuberculosis Treatment Cohort Study
Source: PLOS Glob Public Health. 2024 May 2;4(5):e0003156. doi: 10.1371/journal.pgph.0003156 (PMC11065219; doi:10.1371/journal.pgph.0003156)
Supplement: S2 Table — Abbreviations. BMI, Body mass index; CI, confidence interval; DM, diabetes mellitus; DSSM, direct sputum smear microscopy; HbA1c, glycated hemoglobin, GSIS, Government Service Insurance System; PhilHealth, Philippines Health Insurance, SSS, Republic of the Philippines Social Security Scheme; TB, tuberculosis. Abbreviations. BMI, Body mass index; CI, confidence interval; DM, diabetes mellitus; DSSM, direct sputum smear microscopy; HbA1c, glycated hemoglobin, GSIS, Government Service Insurance System; PhilHealth, Philippines Health Insurance, SSS, Republic of the Philippines Social Security Scheme; TB, tuberculosis. a P-value from Global Wald test with small-sample adjustment for fixed effects [56]. Bold type: P<0.1 for model building and retention criteria. b Results shown for covariates retained in the final multivariable model. c Square root of time, measured as days from start of treatment with an added constant of 0.01. d Any of the following plans: Philippines Health Insurance plan, Social Security, or Government Service Insurance. e BMI according to WHO criteria for adults: underweight (BMI<18.5 kg/m2), normal (BMI 18.5–25.0), overweight (25.0–29.9), obese (BMI >30) [48]. f Normal (Systolic blood pressure (SBP) <120 and diastolic blood pressure (DBP) <80 mm Hg); elevated (SBP 120–129 mm Hg and DBP <80 mm Hg); Stage 1 Hypertension (SBP 130–139 mm Hg and DBP 80–89 mm Hg); and Stage 2 hypertension (SBP > 140 mm Hg and DBP > 90 mm Hg), by the 2017 American College of Cardiology and American Heart Association guidelines [50]. g Based on waist-to-hip ratio >0.85 for women and >0.9 for men used by the WHO for use in diagnostic criteria for metabolic syndrome [49]. h Time-varying exposure; medication (any or individual) use reported at appointment. i After enrollment in TB treatment, report of experiencing any of the TANDEM study DM complications (Ugarte-Gil et al. 2020): ever lost a limb or digit not through trauma, ever had a bypass or stenting surgery in limbs, non- [file pgph.0003156.s007.docx]

**S2 Table.** Unadjusted Associations with HbA1c (%) During TB Treatment Among 188 TB Patients with a DM Comorbidity at 13 Public TB-DOTS Clinics in Metro Manila, Cebu and Negros Occidental, Philippines, 2018-2021

| Characteristic | Participants | HbA1c observations | Coefficient (95% CI) | P-value^a^ | Time interaction  p-value^b^ |
| --- | --- | --- | --- | --- | --- |
| Time since start of TB treatment^c^ | 188 | 482 | -0.08 (-0.109, -0.489) | <0.001 |  |
| Age | 188 | 482 | 0.00 (-0.025, 0.029) | 0.90 |  |
| Sex |  | 482 |  | 0.21 | 0.66 |
| Female | 57 |  | Reference |  |  |
| Male | 131 |  | 0.48 (-0.255, 1.223) |  |  |
| Region |  | 482 |  | 0.02 | 0.22 |
| Manila | 28 |  | Reference |  |  |
| Negros Occidental | 77 |  | 0.28 (-0.442, 1.000) |  |  |
| Cebu | 83 |  | 1.46 (0.426, 2.502) |  |  |
| Residential area |  | 482 |  | 0.04 | 0.22 |
| Urban | 48 |  | Reference |  |  |
| Peri-urban | 86 |  | -1.10 (-1.938, -0.268) |  |  |
| Rural | 54 |  | -0.82 (-1.738, 0.101) |  |  |
| Education |  |  |  |  |  |
| Primary school or below | 44 |  | Reference | 0.22 | 0.69 |
| Above primary school | 144 |  | 0.50 (-0.303, 1.299) |  |  |
| Employment status |  | 482 |  | 0.71 | 0.92 |
| Unemployed | 123 |  | Reference |  |  |
| Employed | 65 |  | 0.13 (-0.583, 0.852) |  |  |
| Possess health insurance^d^ |  | 475 |  | 0.83 | 0.35 |
| No | 58 |  | Reference |  |  |
| Yes | 115 |  | 0.08 (-0.621, 0.836) |  |  |
| Annual income |  | 481 |  | 0.34 | 0.26 |
| <5,000 PHP | 73 |  | Reference |  |  |
| 5,000-9,999 PHP | 50 |  | -0.63 ( -1.484, 0.225) |  |  |
| >10,000 PHP | 64 |  | -0.15 (-0.954, 0.659) |  |  |
| Marital status |  | 482 |  | 0.43 | 0.26 |
| Single | 37 |  | Reference |  |  |
| Married | 134 |  | 0.29 (-0.57, 1.16) |  |  |
| Divorced, separated, or widowed | 17 |  | -0.44 (-1.79, 0.92) |  |  |
| Smoking status |  | 482 |  | 0.82 | 0.31 |
| Never smoked | 94 |  | Reference |  |  |
| Current smoker | 32 |  | -0.30 (-1.26, 0.65) |  |  |
| Ex-smoker | 62 |  | -0.05 (-0.82, 0.71) |  |  |
| BMI classification^e^ |  | 482 |  | 0.27 | 1.00 |
| Normal | 45 |  | Reference |  |  |
| Underweight | 115 |  | -0.67 (-1.48, 0.15) |  |  |
| Overweight or obese | 28 |  | -0.15 (-1.13, 0.83) |  |  |
| Blood pressure^f^ |  | 482 |  | <0.01 | 0.94 |
| Normal | 74 |  | Reference |  |  |
| Elevated | 16 |  | -1.12 (-2.40, 0.16) |  |  |
| Stage 1 Hypertension | 47 |  | 0.60 (-0.26, 1.46) |  |  |
| Stage 2 Hypertension | 35 |  | -0.03 (-1.00, 0.93) |  |  |
| Central obesity^g^ |  | 482 |  | <0.01 | 0.09 |
| Normal | 57 |  | Reference |  |  |
| Central obesity | 131 |  | 0.96 (0.23, 1.69) |  |  |
| Timing of DM diagnosis |  | 482 |  | <0.001 | 0.49 |
| Newly diagnosed | 99 |  | Reference |  |  |
| Previously diagnosed | 89 |  | 1.54 (0.89, 2.18) |  |  |
| Current use of metformin, insulin *or* glycazide^h^ |  |  |  | 0.58 | 0.73 |
| No | 214 | 430 | Reference |  |  |
| Yes | 824 |  | 0.11 (-0.31, 0.58) |  |  |
| Current metformin use^h^ |  | 483 |  | 0.62 | 0.62 |
| No | 71 |  | Reference |  |  |
| Yes | 118 |  | 0.11 (-0.32, 0.54) |  |  |
| Current insulin use^h^ |  | 483 |  | 0.51 | 0.99 |
| No | 164 |  | Reference |  |  |
| Yes | 25 |  | 0.28 (-0.55, 1.11) |  |  |
| Current glyazide use^h^ |  | 483 |  | 0.25 | 0.69 |
| No | 160 |  | Reference |  |  |
| Yes | 29 |  | -0.41 (-1.10, 0.29) |  |  |
| Any reported DM complication^i^ |  |  |  |  |  |
| No | 52 |  | Reference | 0.04 | 0.21 |
| Yes | 137 |  | 0.81 (0.26, 1.58) |  |  |
| Any reported lifestyle changes for DM^j^ |  |  |  | 1.00 | 0.06 |
| No | 48 | 483 | Reference |  |  |
| Yes | 140 |  | 0.00 (-0.77, 0.78) |  |  |
| Type of facility |  | 483 |  | 0.93 | 0.02 |
| City Health Center | 82 |  | Reference |  |  |
| Rural Health Unit | 54 |  | 0.06 (-0.88, 0.75) |  |  |
| Public hospital | 52 |  | 0.11 (-0.74, 0.95) |  |  |
| New versus relapse TB case |  | 481 |  | 0.06 | 0.03 |
| New | 119 |  | Reference |  |  |
| Relapse | 41 |  | -0.66 (-1.36, 0.04) |  |  |
| Drug resistance status |  | 483 |  | 0.22 | 0.03 |
| Drug sensitive | 147 |  | Reference |  |  |
| Drug resistant | 41 |  | -0.52 (-1.35, 0.31) |  |  |
| Basis of TB diagnosis^k^ |  | 483 |  | 0.53 | 0.02 |
| Clinically/by symptom | 62 |  | Reference |  |  |
| Bacteriologically confirmed | 126 |  | 0.23 (-0.50, 0.95) |  |  |
| Length of time with TB symptoms before treatment (weeks) | 188 | 481 | -0.01 (-0.5, 0.03) | 0.74 | 0.69 |
| Fever reported at baseline |  | 483 |  | 0.76 | 0.58 |
| No | 111 |  | Reference |  |  |
| Yes | 77 |  | 0.07 (-0.63, 0.76) |  |  |
| Cough reported at baseline |  | 483 |  | 0.02 | 0.72 |
| No | 16 |  | Reference |  |  |
| Yes | 172 |  | -1.46 (-2.68, -0.24) |  |  |
| Fatigue reported at baseline |  | 483 |  | 0.21 | 0.54 |
| No | 71 |  | Reference |  |  |
| Yes | 117 |  | 0.45 (-0.25, 1.15) |  |  |
| Chills reported at baseline |  | 483 |  | 0.83 | 0.56 |
| No | 151 |  | Reference |  |  |
| Yes | 37 |  | -0.10 (-0.96 0.76) |  |  |
| Weight loss reported at baseline |  | 483 |  | 0.73 | 0.04 |
| No | 68 |  | Reference |  |  |
| Yes | 120 |  | 0.12 (-0.59, 0.84) |  |  |
| Reduced appetite reported at baseline |  | 483 |  | 0.74 | 0.67 |
| No | 118 |  | Reference |  |  |
| Yes | 70 |  | 0.12 (-0.58, 0.82) |  |  |
| Hemoptysis reported at baseline |  | 483 |  | 0.51 | 0.16 |
| No | 117 |  | Reference |  |  |
| Yes | 71 |  | -0.23 (-0.93, 0.47) |  |  |
| Night sweats reported at baseline |  |  |  | 0.53 | 0.89 |
| No | 117 |  | Reference |  |  |
| Yes | 71 |  | -0.22 (-0.09, 0.48) |  |  |
| Chest pain reported at baseline |  |  |  | 0.26 | 0.70 |
| No | 116 |  | Reference |  |  |
| Yes | 72 |  | -0.40 (-1.01, 0.30) |  |  |
| Adherence to TB medications^l^ |  | 299 |  | 0.38 | 0.85 |
| Low |  |  | Reference |  |  |
| Medium |  |  | 0.37 (-0.46, 1.20) |  |  |

| Abbreviations. BMI, Body mass index; CI, confidence interval; DM, diabetes mellitus; DSSM, direct sputum smear microscopy; HbA1c, glycated hemoglobin, GSIS, Government Service Insurance System; PhilHealth, Philippines Health Insurance, SSS, Republic of the Philippines Social Security Scheme; TB, tuberculosis | |
| --- | --- |
| ^a^ | P-value from Global Wald test with small-sample adjustment for fixed effects (StataCorp 2021a). Bold type: P<0.1 for model building and retention criteria |
| ^b^ | Results shown for covariates retained in the final multivariable model. |
| ^c^ | Square root of time, measured as days from start of treatment with an added constant of 0.01 |
| ^d^ | Any of the following plans: Philippines Health Insurance plan, Social Security, or Government Service Insurance |
| ^e^ | BMI according to WHO criteria for adults: underweight (BMI<18.5 kg/m^2)^, normal (BMI 18.5–25.0), overweight (25.0-29.9), obese (BMI >30) (World Health Organizaztion 2021). |
| ^f^ | Normal (Systolic blood pressure (SBP) <120 and diastolic blood pressure (DBP) <80 mm Hg); elevated (SBP 120-129 mm Hg and DBP <80 mm Hg); Stage 1 Hypertension (SBP 130-139 mm Hg and DBP 80-89 mm Hg); and Stage 2 hypertension (SBP > 140 mm Hg and DBP > 90 mm Hg), ﻿by the 2017 American College of Cardiology and American Heart Association guidelines (Whelton *et al.* 2018) |
| ^g^ | Based on waist-to-hip ratio >0.85 for women and >0.9 for men used by the WHO for use in diagnostic criteria for metabolic syndrome (World Health Organization 2008). |
| ^h^ | Time-varying exposure; medication (any or individual) use reported at appointment. |
| ^i^ | After enrollment in TB treatment, report of experiencing any of the TANDEM study DM complications (Ugarte-Gil *et al.* 2020): ever lost a limb or digit not through trauma, ever had a bypass or stenting surgery in limbs, non-healing wound for three or more months, heart attack, stroke, bypass or stenting heart surgery, diagnosis of angina or heart failure, cataract or laser eye surgery, glaucoma, acquired blindness not due to trauma, difficulty seeing or disturbed vision, renal failure. Additionally, the measure captures if participant had any symptom of distal symmetrical peripheral neuropathy using the Michigan Neuropathy Screening Instrument (Feldman *et al.* 1994). |
| ^j^ | After enrollment in TB treatment, report of any of the following lifestyle changes for DM management: changes in eating habits, becoming more physically active, weight management, cutting back or quitting smoking, management stress, or decreasing alcohol intake. |
| ^k^ | Confirmed by GeneXpert (Cepheid), a cartridge-based nucleic acid amplification test for simultaneous rapid tuberculosis diagnosis and rapid antibiotic sensitivity test, or by direct sputum smear microscopy. |
| ^l^ | Adherence to TB treatment (repeated measure) determined by number of affirmative responses to an eight-question Morisky Medication Adherence Scale (MMAS-8). High adherence = 8 affirmative responses, medium adherence = 6-7, low adherence <6. No participants had a “High” MMAS score. 214 missing observations. |
|  |  |
