# Supplementary material for: Glycemic control during TB treatment among Filipinos: The Starting Anti-Tuberculosis Treatment Cohort Study
Source: PLOS Glob Public Health. 2024 May 2;4(5):e0003156. doi: 10.1371/journal.pgph.0003156 (PMC11065219; doi:10.1371/journal.pgph.0003156)
Supplement: S3 Table — Abbreviations: Tuberculosis (TB), Philippine peso (PHP), Hypertension (HT), Body Mass Index (BMI), Confidence interval (CI), Direct sputum smear microscopy (DSSM). a Amongst those with > 2 HbA1c results: Uncontrolled (at least two study-measured HbA1c results equal to or greater than 8%); controlled (at least two study-measured HbA1c results less than 8%). Binary glycemic control outcome is not mutually exclusive to degree of glycemic control outcome. b Amongst those with > 3 HbA1c results: Controlled (all HbA1c values were less than 8%); initially-uncontrolled (baseline HbA1c measurement was greater than 8%, and all subsequent measurements were less than 8%); consistently-uncontrolled (all HbA1c values were equal to or greater than 8%). Degree of glycemic control outcome is not mutually exclusive with binary glycemic control outcome. c Relative risk ratio, which approximates an odds ratio when there are more than two mutually exclusive categories [57,59]. d Any of the following plans: Philippines Health Insurance plan, Social Security, or Government Service Insurance. e BMI according to WHO criteria for adults: underweight (BMI<18.5 kg/m2), normal (BMI 18.5–25.0), overweight (25.0–29.9), obese (BMI >30) [48]. f Normal (Systolic blood pressure (SBP) <120 and diastolic blood pressure (DBP) <80 mm Hg); elevated (SBP 120–129 mm Hg and DBP <80 mm Hg); Stage 1 Hypertension (SBP 130–139 mm Hg and DBP 80–89 mm Hg); and Stage 2 hypertension (SBP > 140 mm Hg and DBP > 90 mm Hg), by the 2017 American College of Cardiology and American Heart Association guidelines [50]. g Based on waist-to-hip ratio >0.85 for women and >0.9 for men used by the WHO for use in diagnostic criteria for metabolic syndrome [49]. h Self-reported at enrollment or point during TB treatment. i After enrollment in TB treatment, report of experiencing any of the TANDEM study DM complications [52]: ever lost a limb or digit not through trauma, ever had a bypass or stenting surgery in limbs, non-healing wou [file pgph.0003156.s008.docx]

**S3 Table.** Unadjusted Associations with Uncontrolled Blood Glucose During TB Treatment among a Subset of 188 TB Patients with a DM Comorbidity at 13 Public TB-DOTS clinics in Metro Manila, Cebu and Negros Occidental, Philippines, 2018-2021

|  |  | **Logistic regression:**  **Uncontrolled versus controlled^a^** | | |  | **Multinomial regression:**  **Initially-uncontrolled versus controlled^b^** | | | **Multinomial regression:**  **Consistently-uncontrolled versus controlled^b^** | | | |  |
| --- | --- | --- | --- | --- | --- | --- | --- | --- | --- | --- | --- | --- | --- |
| Characteristic | Total | Uncontrolled  (%) | Odds Ratio  (95% CI) | Wald P-value | Total | Initially-uncontrolled (%) | Relative Risk Ratio^c^  (95% CI) | Wald P-value | | Consistently uncontrolled  (%) | Relative Risk Ratio^c^ (95% CI) | Wald P-value | |
| Age (years) | 151 | 66 (43.7) | 1.00 (0.97, 1.03) | 1.00 | 113 | 27 (23.9) | 1.03 (0.99, 1.07) | 0.27 | | 33 (29.2) | 0.99 (0.96, 1.03) | 0.27 | |
| Sex |  |  |  | 0.08 |  |  |  | 0.03 | |  |  |  | |
| Female | 46 | 25 (54.3) | Reference |  | 32 | 4 (12.5) | Reference |  | | 15 (46.9) | Reference | 0.03 | |
| Male | 105 | 41 (39.0) | 0.54 (0.27, 1.08) |  | 81 | 23 (28.4) | 1.87 (0.54, 6.41) |  | | 18 (22.2) | 0.39 (0.15, 0.99) |  | |
| Region |  |  |  | 0.01 |  |  |  | 0.83 | |  |  |  | |
| Cebu | 70 | 27 (38.6) | Reference |  | 55 | 13 (23.6) | Reference |  | | 16 (29.1) | Reference | 0.83 | |
| Negros Occidental | 66 | 26 (39.4) | 1.04 (0.52, 2.06) |  | 54 | 12 (22.2) | 0.92 (0.36, 2.40) |  | | 16 (29.6) | 1.00 (0.41, 2.41) |  | |
| Manila | 15 | 13 (86.7) | 10.35 (2.17, 49.49) |  | 4 | 2 (50.0) | 4.00 (0.33, 48.30) |  | | 1 (25.0) | 1.62 (0.09, 27.84) |  | |
| Location |  |  |  | 0.10 |  |  |  | 0.20 | |  |  |  | |
| Urban | 30 | 18 (60.0) | Reference |  | 17 | 8 (47.1) | Reference |  | | 4 (23.5) | Reference | 0.20 | |
| Peri-urban | 76 | 28 (36.8) | 0.39 (0.16, 0.92) |  | 61 | 13 (21.3) | 0.26 (0.07, 0.95) |  | | 17 (27.9) | 0.69 (0.16, 2.90) |  | |
| Rural | 45 | 20 (44.4) | 0.53 (0.21, 1.36) |  | 35 | 6 (17.1) | 0.22 (0.05, 0.94) |  | | 12 (34.3) | 0.88 (0.20, 3.99) |  | |
| Absolute household income |  |  |  | 0.75 |  |  |  | 0.47 | |  |  |  | |
| Less than 5,000 PHP | 63 | 29 (46.0) | Reference |  | 48 | 10 (20.8) | Reference |  | | 16 (33.3) | Reference | 0.47 | |
| 5000, 9999 PHP | 39 | 15 (38.5) | 0.73 (0.32, 1.65) |  | 31 | 6 (19.4) | 0.73 (0.22, 2.41) |  | | 7 (22.6) | 0.53 (0.18, 1.58) |  | |
| >10,000 PHP | 48 | 21 (43.8) | 0.91 (0.43, 1.94) |  | 34 | 11 (32.4) | 1.86 (0.62, 5.58) |  | | 10 (29.4) | 1.06 (0.37, 3.01) |  | |
| Marital status |  |  |  | 0.13 |  |  |  | 0.14 | |  |  |  | |
| Single | 29 | 10 (34.5) | Reference |  | 20 | 1 (5.0) | Reference |  | | 5 (25.0) | Reference | 0.14 | |
| Married | 109 | 53 (48.6) | 1.80 (0.77, 4.22) |  | 82 | 20 (24.4) | 8.23 (1.01, 67.35) |  | | 28 (34.1) | 2.31 (0.74, 7.19) |  | |
| Divorced, separated or widowed | 13 | 3 (23.1) | 0.57 (0.13, 2.56) |  | 11 | 6 (54.5) | 16.78 (1.60, 175.95) |  | | 0 (0.0) | 0.00 (0.00, .) |  | |
| Employment status |  |  |  | 0.92 |  |  |  | 0.85 | |  |  |  | |
| Unemployed | 100 | 44 (44.0) | Reference |  | 78 | 18 (23.1) | 1 |  | | 22 (28.2) | Reference | 0.85 | |
| Employed | 51 | 22 (43.1) | 0.97 (0.49, 1.91) |  | 35 | 9 (25.7) | 1.27 (0.47, 3.44) |  | | 11 (31.4) | 1.27 (0.50, 3.24) |  | |
| Possess health insurance^d^ |  |  |  | 0.52 |  |  |  | 0.14 | |  |  |  | |
| No | 45 | 18 (40.0) | Reference |  | 35 | 4 (11.4) | 1 |  | | 11 (31.4) | Reference | 0.14 | |
| Yes | 94 | 43 (45.7) | 1.26 (0.61, 2.60) |  | 69 | 19 (27.5) | 3.39 (1.00, 11.51) |  | | 22 (31.9) | 1.43 (0.57, 3.60) |  | |
| Smoking status |  |  |  | 0.19 |  |  |  | 0.21 | |  |  |  | |
| No smoking experience | 75 | 38 (50.7) | Reference |  | 52 | 9 (17.3) | Reference |  | | 19 (36.5) | Reference | 0.21 | |
| Current smoker | 26 | 11 (42.3) | 0.71 (0.29, 1.76) |  | 20 | 4 (20.0) | 1.19 (0.29, 4.83) |  | | 7 (35.0) | 0.98 (0.31, 3.12) |  | |
| Ex-smoker | 50 | 17 (34.0) | 0.50 (0.24, 1.05) |  | 41 | 14 (34.1) | 1.87 (0.67, 5.21) |  | | 7 (17.1) | 0.44 (0.15, 1.26) |  | |
| BMI classification at baseline^e^ |  |  |  | 0.02 |  |  |  | 0.04 | |  |  |  | |
| Normal | 91 | 42 (46.2) | Reference |  | 61 | 15 (24.6) | Reference |  | | 22 (36.1) | Reference | 0.04 | |
| Underweight | 36 | 9 (25.0) | 0.39 (0.16, 0.92) |  | 32 | 4 (12.5) | 0.29 (0.08, 1.01) |  | | 6 (18.8) | 0.30 (0.10, 0.87) |  | |
| Overweight/obese | 24 | 15 (62.5) | 1.94 (0.77, 4.90) |  | 20 | 8 (40.0) | 1.83 (0.55, 6.08) |  | | 5 (25.0) | 0.78 (0.22, 2.82) |  | |
| Blood pressure at baseline^f^ |  |  |  | 0.08 |  |  |  | 0.35 | |  |  |  | |
| Normal | 61 | 22 (36.1) | Reference |  | 46 | 8 (17.4) | Reference |  | | 11 (23.9) | Reference | 0.35 | |
| Elevated | 12 | 3 (25.0) | 0.59 (0.14, 2.41) |  | 10 | 3 (30.0) | 2.02 (0.40, 10.38) |  | | 2 (20.0) | 0.98 (0.17, 5.84) |  | |
| Stage 1 Hypertension | 43 | 25 (58.1) | 2.46 (1.11, 5.48) |  | 33 | 11 (33.3) | 3.71 (1.16, 11.89) |  | | 12 (36.4) | 2.95 (0.99, 8.79) |  | |
| Stage 2 Hypertension | 19 | 9 (47.4) | 1.60 (0.56, 4.52) |  | 13 | 4 (30.8) | 2.25 (0.51, 9.99) |  | | 3 (23.1) | 1.23 (0.26, 5.80) |  | |
| Central obesity^g^ |  |  |  | <0.01 |  |  |  | <0.01 | |  |  |  | |
| No | 49 | 12 (24.5) | Reference |  | 39 | 3 (7.7) | Reference |  | | 8 (20.5) | Reference | <0.01 | |
| Yes | 102 | 54 (52.9) | 3.47 (1.62, 7.41) |  | 74 | 24 (32.4) | 8.96 (2.40, 33.40) |  | | 25 (33.8) | 3.50 (1.34, 9.16) |  | |
| Timing of DM diagnosis |  |  |  | <0.001 |  |  |  | <0.001 | |  |  |  | |
| Newly diagnosed | 90 | 26 (28.9) | Reference |  | 71 | 14 (19.7) | Reference |  | | 11 (15.5) | Reference | <0.001 | |
| Previously diagnosed | 61 | 40 (65.6) | 4.69 (2.33, 9.42) |  | 42 | 13 (31.0) | 6.10 (2.04, 18.27) |  | | 22 (52.4) | 13.14 (4.48, 38.52) |  | |
| Report of insulin, metformin *or* insulin during TB treatment^h^ |  |  |  |  |  |  |  |  | |  |  |  | |
| No use | 49 | 7 (14.3) | Reference | <0.001 | 41 | 2 (4.8) | Reference | <0.001 | | 3 (7.3) | Reference | <0.001 | |
| Use | 102 | 59 (57.8) | 8.23 (3.68, 20.08) |  | 72 | 25 (34.7) | 3.05 (1.73, 4.37) |  | | 30 (41.7) | 3.27 (1.72, 4.83) |  | |
| Use of metformin^h^ |  |  |  | <0.001 |  |  |  | <0.001 | |  |  |  | |
| No use | 58 | 12 (20.7) | Reference |  | 112 | 26 (23.2) | Reference |  | | 7 (14.3) | Reference | <0.001 | |
| Use | 93 | 54 (58.1) | 5.31 (2.49, 11.32) |  | 1 | 1 (100.0) | 14.57 (4.31, 49.26) |  | | 26 (40.6) | 9.41 (3.37, 26.26) |  | |
| Use of insulin^h^ |  |  |  | 0.01 |  |  |  | <0.01 | |  |  |  | |
| No use | 132 | 52 (39.4) | Reference |  | 49 | 4 (8.2) | Reference |  | | 22 (22.0) | Reference | <0.01 | |
| Use | 19 | 14 (73.7) | 4.31 (1.46, 12.67) |  | 64 | 23 (35.9) | 2.00 (0.12, 33.27) |  | | 11 (84.6) | 26.00 (3.16, 213.80) |  | |
| Use of gliclazide^h^ |  |  |  | 0.38 |  |  |  | 0.16 | |  |  |  | |
| No use | 128 | 54 (42.2) | Reference |  | 100 | 26 (26.0) | Reference |  | | 25 (26.6) | Reference | 0.16 | |
| Use | 23 | 12 (52.2) | 1.49 (0.61, 3.64) |  | 13 | 1 (7.7) | 2.74 (0.75, 9.99) |  | | 8 (42.1) | 3.07 (0.91, 10.38) |  | |
| Report of DM complication^i^ |  |  |  | 0.05 |  |  |  | 0.17 | |  |  |  | |
| Never | 52 | 8 (28.6) | Reference |  | 94 | 21 (22.3) | Reference |  | | 7 (33.3) | Reference | 0.24 | |
| Yes | 134 | 58 (47.2) | 2.23 (0.91- 5.45) |  | 19 | 6 (31.6) | 4.49 (0.94, 21.45) |  | | 26 (28.9) | 1.14 (0.40, 3.29) |  | |
| Report of lifestyle change for DM^j^ |  |  |  | 0.58 |  |  |  | 0.02 | |  |  |  | |
| Never | 40 | 16 (40.0) | Reference |  | 23 | 2 (8.7) | Reference |  | | 5 (17.2) | Reference | 0.17 | |
| Yes | 111 | 50 (45.0) | 1.23 (0.59, 2.56) |  | 90 | 25 (27.8) | 1.40 (0.55, 3.58) |  | | 28 (33.3) | 2.01 (0.65, 6.23) |  | |
| Type of TB facility |  |  |  | 0.99 |  |  |  | 0.55 | |  |  |  | |
| City Health Center | 71 | 31 (43.7) | Reference |  | 53 | 12 (22.6) | Reference |  | | 15 (28.3) | Reference | 0.55 | |
| Rural Health Unit | 45 | 20 (44.4) | 1.03 (0.49, 2.19) |  | 35 | 6 (17.1) | 0.76 (0.24, 2.43) |  | | 12 (34.3) | 1.22 (0.46, 3.24) |  | |
| Public hospital | 35 | 15 (42.9) | 0.97 (0.43, 2.19) |  | 25 | 9 (36.0) | 1.95 (0.63, 6.04) |  | | 6 (24.0) | 1.04 (0.31, 3.44) |  | |
| Basis of TB diagnosis^k^ |  |  |  | 0.92 |  |  |  | 0.05 | |  |  |  | |
| Clinically diagnosed | 51 | 22 (43.1) | Reference |  | 39 | 4 (10.3) | Reference |  | | 15 (38.5) | Reference | 0.05 | |
| Bacteriologically confirmed | 100 | 44 (44.0) | 1.04 (0.52, 2.05) | 0.56 | 74 | 23 (31.1) | 3.48 (1.05, 11.55) | 0.64 | | 18 (24.3) | 0.73 (0.30, 1.76) |  | |
| Duration of TB symptoms before diagnosis (weeks) | 188 | 66 (43.7) | 0.99 (0.94, 1.03) |  | 113 | 27 (23.9) | 0.99 (0.93, 1.06) |  | |  | 0.97 (0.90, 1.04) | 0.64 | |
| New versus relapse TB case |  |  |  | 0.03 |  |  |  | 0.05 | |  |  |  | |
| New | 96 | 48 (50.0) | Reference |  | 72 | 15 (20.8) | Reference | 33 (29.2) | | 27 (37.5) | Reference | 0.05 | |
| Relapse | 54 | 17 (31.5) | 0.46 (0.23, 0.93) |  | 41 | 12 (29.3) | 1.04 (0.41, 2.65) |  | | 6 (14.6) | 0.29 (0.10, 0.82) |  | |
| Type of TB infection |  |  |  | 0.08 |  |  |  | 0.58 | |  |  |  | |
| Drug sensitive | 118 | 56 (47.5) | Reference |  | 87 | 19 (21.8) | Reference |  | | 27 (31.0) | Reference | 0.58 | |
| Drug resistant | 33 | 10 (30.3) | 0.48 (0.21, 1.10) |  | 26 | 8 (30.8) | 1.44 (0.50, 4.10) |  | | 6 (23.1) | 0.76 (0.25, 2.27) |  | |
| Median adherence to TB treatment^l^ |  |  |  | 0.75 |  |  |  | 1.00 | |  |  |  | |
| Medium | 145 | 63 (43.4) | Reference |  | 112 | 26 (23.2) | Reference |  | | 33 (29.5) | Reference | 1.00 | |
| Low | 6 | 3 (50.0) | 1.30 (0.25, 6.67) |  | 1 | 1 (100.0) | 0.0 (0.00, 0.00) |  | | NA | NA |  | |
| Cough at baseline |  |  |  | 0.06 |  |  |  | 0.37 | |  |  |  | |
| No | 11 | 8 (72.7) | Reference |  | 8 | 2 (25.0) | Reference |  | | 4 (50.0) | Reference | 0.37 | |
| Yes | 140 | 58 (41.4) | 0.27 (0.07, 1.04) |  | 105 | 25 (23.8) | 0.49 (0.07, 3.69) |  | | 29 (27.6) | 0.28 (0.05, 1.65) |  | |
| Fever at baseline |  |  |  | 0.61 |  |  |  | 0.65 | |  |  |  | |
| No | 88 | 40 (45.5) | Reference |  | 67 | 17 (25.4) | Reference |  | | 21 (31.3) | Reference | 0.65 | |
| Yes | 63 | 26 (41.3) | 0.84 (0.44, 1.62) |  | 46 | 10 (21.7) | 0.71 (0.27, 1.84) |  | | 12 (26.1) | 0.69 (0.28, 1.68) |  | |
| Fatigue at baseline |  |  |  | 0.37 |  |  |  | 0.78 | |  |  |  | |
| No | 61 | 24 (39.3) | Reference |  | 48 | 10 (20.8) | Reference |  | | 14 (29.2) | Reference | 0.78 | |
| Yes | 90 | 42 (46.7) | 1.35 (0.70, 2.61) |  | 65 | 17 (26.2) | 1.41 (0.54, 3.64) |  | | 19 (29.2) | 1.12 (0.47, 2.70) |  | |
| Chills at baseline |  |  |  | 0.60 |  |  |  | 0.22 | |  |  |  | |
| No | 123 | 55 (44.7) | Reference |  | 92 | 19 (20.7) | Reference |  | | 29 (31.5) | Reference | 0.22 | |
| Yes | 28 | 11 (39.3) | 0.80 (0.35, 1.85) |  | 21 | 8 (38.1) | 2.06 (0.69, 6.15) |  | | 4 (19.0) | 0.67 (0.19, 2.40) |  | |
| Night sweats at baseline |  |  |  | 0.28 |  |  |  | 0.61 | |  |  |  | |
| No | 91 | 43 (47.3) | Reference |  | 67 | 15 (22.4) | Reference |  | | 18 (26.9) | Reference | 0.61 | |
| Yes | 60 | 23 (38.3) | 0.69 (0.36, 1.35) |  | 46 | 12 (26.1) | 1.43 (0.56, 3.68) |  | | 15 (32.6) | 1.49 (0.61, 3.62) |  | |
| Reduced appetite at baseline |  |  |  | 0.71 |  |  |  | 0.74 | |  |  |  | |
| No | 94 | 40 (42.6) | Reference |  | 71 | 18 (25.4) | Reference |  | | 19 (26.8) | Reference | 0.74 | |
| Yes | 57 | 26 (45.6) | 1.13 (0.58, 2.20) |  | 42 | 9 (21.4) | 0.89 (0.34, 2.38) |  | | 14 (33.3) | 1.32 (0.54, 3.21) |  | |
| Chest pain at baseline |  |  |  | 0.16 |  |  |  | 0.29 | |  |  |  | |
| No | 91 | 44 (48.4) | Reference |  | 68 | 19 (27.9) | Reference |  | | 21 (30.9) | Reference | 0.29 | |
| Yes | 60 | 22 (36.7) | 0.62 (0.32, 1.20) |  | 45 | 8 (17.8) | 0.47 (0.18, 1.26) |  | | 12 (26.7) | 0.64 (0.26, 1.56) |  | |
| Weight loss at baseline |  |  |  | 0.93 |  |  |  | 0.94 | |  |  |  | |
| No | 52 | 23 (44.2) | Reference |  | 37 | 9 (24.3) | Reference |  | | 10 (27.0) | Reference | 0.94 | |
| Yes | 99 | 43 (43.4) | 0.97 (0.49, 1.90) |  | 76 | 18 (23.7) | 1.03 (0.39, 2.74) |  | | 23 (30.3) | 1.18 (0.46, 3.01) |  | |
| Hemoptysis at baseline |  |  |  | 0.37 |  |  |  | 0.97 | |  |  |  | |
| No | 90 | 42 (46.7) | Reference |  | 63 | 15 (23.8) | Reference |  | | 19 (30.2) | Reference | 0.97 | |
| Yes | 61 | 24 (39.3) | 0.74 (0.38, 1.43) |  | 50 | 12 (24.0) | 0.97 (0.38, 2.46) |  | | 14 (28.0) | 0.89 (0.37, 2.14) |  | |

| Abbreviations: Tuberculosis (TB), Philippine peso (PHP), Hypertension (HT), Body Mass Index (BMI), Confidence interval (CI), Direct sputum smear microscopy (DSSM) | |
| --- | --- |
| ^a^ | Amongst those with > 2 HbA1c results: Uncontrolled (at least two study-measured HbA1c results equal to or greater than 8%); controlled (at least two study-measured HbA1c results less than 8%). Binary glycemic control outcome is not mutually exclusive to degree of glycemic control outcome |
| ^b^ | Amongst those with > 3 HbA1c results: Controlled (all HbA1c values were less than 8%); initially-uncontrolled (baseline HbA1c measurement was greater than 8%, and all subsequent measurements were less than 8%); consistently-uncontrolled (all HbA1c values were equal to or greater than 8%). Degree of glycemic control outcome is not mutually exclusive with binary glycemic control outcome. |
| ^c^ | Relative risk ratio, which approximates an odds ratio when there are more than two mutually exclusive categories (UCLA: Statistical Consulting Group; Gutierrez 2005; StataCorp 2021b) |
| ^d^ | Any of the following plans: Philippines Health Insurance plan, Social Security, or Government Service Insurance |
| ^e^ | BMI according to WHO criteria for adults: underweight (BMI<18.5 kg/m^2)^, normal (BMI 18.5–25.0), overweight (25.0-29.9), obese (BMI >30) (World Health Organizaztion 2021). |
| ^f^ | Normal (Systolic blood pressure (SBP) <120 and diastolic blood pressure (DBP) <80 mm Hg); elevated (SBP 120-129 mm Hg and DBP <80 mm Hg); Stage 1 Hypertension (SBP 130-139 mm Hg and DBP 80-89 mm Hg); and Stage 2 hypertension (SBP > 140 mm Hg and DBP > 90 mm Hg), ﻿by the 2017 American College of Cardiology and American Heart Association guidelines (Whelton *et al.* 2018) |
| ^g^ | Based on waist-to-hip ratio >0.85 for women and >0.9 for men used by the WHO for use in diagnostic criteria for metabolic syndrome (World Health Organization 2008). |
| ^h^ | Self-reported at enrollment or point during TB treatment |
| ^i^ | After enrollment in TB treatment, report of experiencing any of the TANDEM study DM complications (Ugarte-Gil *et al.* 2020): ever lost a limb or digit not through trauma, ever had a bypass or stenting surgery in limbs, non-healing wound for three or more months, heart attack, stroke, bypass or stenting heart surgery, diagnosis of angina or heart failure, cataract or laser eye surgery, glaucoma, acquired blindness not due to trauma, difficulty seeing or disturbed vision, renal failure. Additionally, the measure captures if participant had any symptom of distal symmetrical peripheral neuropathy using the Michigan Neuropathy Screening Instrument (Feldman *et al.* 1994). |
| ^j^ | After enrollment in TB treatment, report of any of the following lifestyle changes for DM management: changes in eating habits, becoming more physically active, weight management, cutting back or quitting smoking, management stress, or decreasing alcohol intake. |
| ^k^ | By DSSM or GeneXpert |
| ^l^ | Adherence to TB treatment (repeated measure) determined by number of affirmative responses to an eight-question Morisky Medication Adherence Scale (MMAS-8). High adherence = 8 affirmative responses, medium adherence = 6-7, low adherence <6. No participants had a “High” MMAS score. 15/188 participants had no MMAS score recorded . |
