# Supplementary material for: Glycemic control during TB treatment among Filipinos: The Starting Anti-Tuberculosis Treatment Cohort Study
Source: PLOS Glob Public Health. 2024 May 2;4(5):e0003156. doi: 10.1371/journal.pgph.0003156 (PMC11065219; doi:10.1371/journal.pgph.0003156)
Supplement: Attachment — Submitted filename: Response to Reviewers 01 26 2024_FINAL.pdf [file pgph.0003156.s009.pdf]

Dr Tansy Edwards
Associate Professor in Biostatistics & Epidemiology

+44 (0) 7927 2825
Tansy.Edwards@lshtm.ac.uk

London School of Hygiene & Tropical Medicine
Keppel Street, London WC1E 7HT
+44 (0)20 7636 8636 • www.lshtm.ac.uk

26 February 2024
PGPH-D-23-01570
Glycemic control during TB treatment among Filipinos: the Starting Anti-Tuberculosis Treatment Cohort Study
PLOS Global Public Health

Dear Dr Wejse,

Please accept submission of our manuscript, which has been revised following peer review. We are very grateful for the insightful comments offered by our reviewers. We provide our response to each comment from the reviewers, below.

General:

1. We understand that your data availability statement currently reads as follows: "Analyses utilized data from the Starting Anti-TB Treatment (St-ATT) cohort (ISRCTN16347615). Anonymized data set should be made available on reasonable request. It will be deposited in LSHTM data compass with the following DOI: ttps://doi.org/10.17037/DATA. 00002476." Please respond to the following prompts:
   a.) The DOI provided is not functional. Please provide a working DOI link to your data repository.
   Response. We have corrected the DOI link for the data repository: https://doi.org/10.17037/DATA.00002313

   b.) If there are ethical or legal restrictions on sharing a de-identified data set, please explain them in detail (e.g., data contain potentially identifying or sensitive patient information, data are owned by a third-party organization, etc.) and who has imposed them (e.g., a Research Ethics Committee or Institutional Review Board, etc.). Please also provide non-author contact information for a data access committee, ethics committee, or other institutional body to which data requests may be sent.
   Response: A minimised anonymised dataset will be made freely available as a dataset linked to the project found at the DOI link, once the manuscript is published.

2. The article does not adhere to appropriate reporting guidelines (STROBE). A completed STROBE checklist should be submitted with the revised manuscript, including a readable flow chart according to STROBE guidelines.
   Response: We have included a STROBE Checklist with the Supporting Information.
   We have edited the flow chart figure and confirmed that it has a clear resolution and meets journal requirements.

3. Please include a complete copy of PLOS' questionnaire on inclusivity in global research in your revised manuscript.

Response: We have included PLOS's questionnaire on inclusivity in global research in our revised manuscript, included with the Supporting Information.

<u>**Reviewer 1:**</u> We thank Reviewer 1 for their thoughtful comments, which have improved the manuscript. We have taken your overall comments on board, including suggestions to make the manuscript more concise and include relevant information for clarity.

1. The report was complete and well-written, but can be made more concise by reducing redundant sentences.
   Response: We have edited the paper throughout to reduce redundancies.

Introduction

2. I suggest removing "during tuberculosis treatment" and "comorbidity" to make the title more concise. "Glycemic control among people with diabetes mellitus in the Starting Anti-Tuberculosis Treatment Cohort Study in the Philippines"
   Response: Thank you for this suggestion; we have simplified the title to reflect your recommendation.

3. The introduction is one and a half pages long. I suggest restricting the introduction to a background (justification for the study conducted) and the aim of the study. Some parts of the introduction may be more suitable under the discussion section.
   Response: We have moved content from the introduction to the discussion.

Methods

4. I suggest removing Tables 1 and 2 and presenting the information in those tables as paragraphs to save some space and make it less like a student's thesis with a shorter description.
   Response: We have tried to reduce the word count and maintain the ease of reading and the clarity. If we maintain table 1 and 2, the word count is reduced and we feel that the information in Tables 1 and 2 is easier to read and reference in tabular format for most readers.

5. Table 1: Please explain why people with "HbA1c <6.5% at baseline and no subsequent HbA1c test exceeds 10%" are categorised as having transient hyperglycaemia.
   Response: We thank the reviewer for their close attention to these definitions. In the absence of official guidance on how to determine transient hyperglycaemia among TB patients using HbA1c data, the definition applied in this analysis was developed by the researchers, drawing from global TB-DM treatment guidelines from the Global Union Against TB and Lung Disease.

   We wanted our definition to capture temporarily elevated blood glucose following the initiation of TB treatment among patients not previously known to be diabetic (e.g., HbA1c <6.5% at enrolment). We determined that patients with a **single** elevated blood glucose result following the start of TB treatment below the Union definition for severe hyperglycaemia (10% during treatment) was indicative of a stress-induced increase in blood glucose levels.

   We have added a footnote to Table 1 to explain this.

6. Ethical approval (line 106): Please add information regarding informed consent.
   Response: We added additional language to the manuscript: St-ATT research nurses completed informed consent procedures—reading consent forms verbally—in quiet and private locations to ensure patients had free and voluntary consent uninfluenced by health center staff.

7. Lines 142-144: Please explain why you are using WHO criteria for BMI instead of the Asia-Pacific criteria (Yoon KH, et al. Epidemic obesity and type 2 diabetes in Asia. Lancet 2006;368(9548):1681–1688).
   Response: We have used the WHO criteria for BMI rather than the Asia-specific criteria to harmonize our sub-study with our parent study.
   Cox, S. E., Edwards, T., Faguer, B. N., Ferrer, J. P., Suzuki, S. J., Koh, M., Ferdous, F., Saludar, N. R., Garfin, A. C. G., Castro, M. C., & Solon, J. A. (2021). Patterns of non-communicable comorbidities at start of tuberculosis treatment in three regions of the Philippines: The St-ATT cohort. PLOS global public health, 1(11), e0000011.

Results

8. Results. The figures were blurry, especially Figure 1.
   Response: We changed Figure 1 to ensure it has proper resolution, and checked all other figures to confirm that they have clear resolution and meet journal requirements. The PDF preview of the manuscript shared with reviewers included low-resolution renderings of the figure.

9. The results section contains many long and hard-to-read sentences.
   Response: We have reviewed the paper carefully and made minor edits, and would be happy to review any specific sentences.

10. Table 3: There are mean and median age; choose one according to the data distribution.
    Response: Age is normally distributed; we have removed median from the table as advised.

11. Table 3: Some of the cells have numbers of less than 5. You may want to consider using the Fisher Exact test rather than the Chi-squared test.
    Response: Thank you for this suggestion. We have revised the paper to use Fisher's exact test instead of the Chi-squared test.

12. Table 3: It is rather surprising that only almost 30% of the patients did not take any glucose-lowering medications during TB treatment.
    Response: We observed that patients who did not ever take a glucose-lowering medication were those who were newly diagnosed and likely did not need pharmaceutical intervention. In this group most (51) of the 55 patient who never reported taking a DM medication were diagnosed with DM at the start of treatment through blood glucose screening. Only 8 of those 51 patients had an HbA1c >8% at their baseline assessment; most experienced at least one more high HbA1c reading during treatment and likely were patients who should have initiated a glucose-lowering medication during treatment.

13. I suggest including Tables S2 and S3 in the result section to understand the results of the univariable analyses before going into the model (Table 4).
    Response: Thank you for this suggestion – we are happy that these tables are useful in understanding the model results. We request to keep the tables in supplementary materials

as they are quite large and the interested reader may easily access them as referenced supplementary materials.

14. Table 4: Please explain why glucose-lowering medication is not included in the model.
Response: The association between the outcome (HbA1c) and use of glucose-lowering medications was not significant in univariable testing (Wald test P-value = 0.58, Supplementary Table 3) and was therefore not used in the stepwise selection of variables to develop the multivariate model.

15. Table 4. Are HbA1c levels at any point in treatment during treatment in Table 4 calculated as mean?
Response. We are not sure what the reviewer is asking.

16. Table 5: the relative risk in the multinomial regression: consistently-uncontrolled versus controlled for those who use glucose-lowering medications is 10.24 (0.07, 0.95). This is likely a typo. Please check.
Response: We are very grateful for our reviewer's close attention to the results. This was indeed a typo and it has been corrected. We have checked all results to ensure accuracy.

Discussion

17. I suggest removing the first paragraph in the discussion section (lines 412-419). The content of this paragraph belongs to the Introduction part or later part of the discussion section. The first paragraph of the discussion section should describe the main result of the paper.
Response: We have deleted most of the first paragraph, and moved some content to the introduction.

18. Lines 472-476 "The logistic regressions found relationships (P<0.05) between uncontrolled blood glucose and glucose-lowering medication use, region, sex, and new versus relapse TB case, which were not observed in the repeated HbA1c analysis. The analysis of repeated HbA1c measures did not identify a relationship with glucose-lowering medication use." To which part of the result does this sentence interpret?
Response: We have edited this sentence so that it clearly refers to the mixed-effects linear regression.

19. There are many redundancies in the results section. I suggest synchronising the statistical analyses presented in the Methods section with the Results section.
Response: We have reviewed the results and methods sections and tried to align the content to reduce redundancies.

20. Too many tables and figures were presented, resulting in a mixed message of the study. You may want to reduce the tables/figures and clarify the main message.
Response: Based on previous feedback, tabular presentation of the information in Tables 1 and 2 in the methods was easier to read. Table 3 is description of the cohort which is standard. Table 4 and 5 are the main adjusted findings for the multivariable analyses.

    Figure 1 is a standard flowchart. The original Figure 2 has been moved to supplementary materials. Figures 3 and 4 are intended to help the reader understand the interaction results and have also been moved to the supplementary materials. With the revisions, there are now 3 results tables and 2 figures in the main body of the paper.

Conclusion

21. Lines 521-523: "Evidence from this study suggests that use of glucose-lowering medication may not be effective during TB treatment or simply an indicator of more severe disease." This conclusion contradicts the results shown in Table 5 and the study limitations (lines 496-499 and lines 504-509). Glucose-lowering medication may be an indication of more severe disease in routine practice and may be less effective during TB treatment unless more intensive monitoring and DM treatment adjustments are implemented (Reference number 44: Ruslami R, Koesoemadinata RC, Soetedjo NNM, Imaculata S, Gunawan Y, Permana H, et al. The effect of a structured clinical algorithm on glycemic control in patients with combined tuberculosis and diabetes in Indonesia: A randomized trial. Diabetes Res Clin Pract 2021;173:108701).
    Response: Thank you for this insightful comment. We have revised our conclusion accordingly.

Reviewer 2: We thank Reviewer 2 for their insightful comments. We have revised the manuscript to address your feedback and suggestions.

Rationale of the study

1. While the introduction mentions the limited published data from Southeast Asia, especially the Philippines, it could provide more explicit information about the specific research gap that the current study aims to address.
   Response: We have added more information about the specific research gap our study aims to address in the introduction.
2. There have been several studies powered researchers conducted on this topic previously compared to your underpowered study. What distinguishes our study from the ones mentioned in the links below?
   a. https://www.ncbi.nlm.nih.gov/pmc/articles/PMC7238456/
   b. https://journals.plos.org/plosmedicine/article?id=10.1371/journal.pmed.1002072
   c. https://www.sciencedirect.com/science/article/pii/S2405579423000244

   Response. The papers you provided all represent valuable contributions to this field of study. We believe that this paper offers a unique contribution. Our study provides a more detailed and complex analysis of blood glucose control among TB-DM patients in a similar theme to Adane, et al. (2023). In addition, our paper explores glycaemic control in a different setting than Adane, et al. (2023), which was based in Ethiopia. Gurukartick, et al. (2020) did not assess factors associated with glycemic control during TB treatment and association between glycamic control and TB treatment outcomes because glycemic control assessment, if any, was based mostly on RBG values. Finally, Lee et al. (2016) examined risk of TB among diabetes patients, using a single fasting blood glucose measurement collected through a DM screening, and did not collect blood glucose measurements during TB treatment as our study did.

Methods

3. The study includes participants from clinics in different locations (urban, peri-urban, rural) and with different focuses (programmatic management of drug-resistant TB). The variability in clinic settings and TB management programs may introduce confounding variables that could impact the generalizability of the findings. The method should address how the

potential site variability is controlled for in the analysis or whether it is considered as a factor.
Response. We investigated the presence of between-site variation (clustering) by comparing multivariable models with and without a random intercept for site (LRT). There was no evidence of between-site variation (clustering) in the repeated measures regression (LRT p-value 0.24) or in the logistic regression (LRT P-value>1.000). Testing for clustering was not repeated for the multinomial regression as this did not seem necessary.
We have updated our methods and results to include this information.

4. The process of clinic selection remains unclear. Further clarification is needed regarding the specific sampling techniques employed in this study.
   Response. We have added language to our methods section to clarify to readers.

   Study sites were chosen to represent urban, semi-urban, and rural populations with adequate TB cases, as assessed through review of TB clinic records within accessible distance. Selection criteria also included practical considerations such as the proximity of a study laboratory for sample processing. Site participation hinged on the willingness of local health authorities and clinic staff, leading investigators to approach more clinics than necessary to ensure an adequate number of sites were enrolled in all regions.

5. The study notes that more participants in Manila had only one HbA1c measurement compared to other regions, and participants in Negros Occidental had different characteristics compared to Manila and Cebu. This variability in data collection and participant characteristics across regions may impact the generalizability of the results and should be addressed in the discussion.
   Response. Thank you. We have added this statement to the discussion section.
   With a higher percentage of participants in Manila having only a baseline measurement compared to the other two regions, our findings may be more reflective of participants in these two regions. Nevertheless, our recommendations relating to monitoring of diabetes control during TB treatment hold, so that it will be possible to explore how control of diabetes links to TB treatment outcomes.

Discussion

6. While the discussion suggests future research directions, such as collecting data from larger cohorts, it lacks a more detailed exploration of specific research questions that need to be addressed. Providing a more nuanced discussion of potential avenues for future research would enhance the conclusion.
   Response. We have added a paragraph to the discussion, addressing what sample size would be needed to determine whether hyperglycaemia during TB treatment increases the risk of TB treatment failure amongst people with diabetes.

7. The discussion provides suggestions for increased support for individuals with previously-diagnosed DM entering TB treatment and highlights the potential significance of central obesity. However, it could benefit from a more explicit discussion of how these findings could be translated into actionable strategies in clinical practice or public health interventions.
   Response. We have added a paragraph to the discussion:

   "National TB Programs might consider developing guidelines to screen new TB patients for a previous DM diagnosis and other potential risk factors such a central obesity, and provide

identified patients with targeted support such as routine glucose monitoring, health education and adjustment of DM medications. An intervention including these resources was tested in a randomised controlled trial among 150 pulmonary TB-DM patients at outpatient TB clinics in Indonesia, and found that TB-DM patients receiving joint TB-DM care experienced a greater decrease in HbA1c during treatment than patients randomised to receive routine DM management (Rulami et al. 2021)."

(Ruslami R, Koesoemadinata RC, Soetedjo NNM, Imaculata S, Gunawan Y, Permana H, et al. The effect of a structured clinical algorithm on glycemic control in patients with combined tuberculosis and diabetes in Indonesia: A randomized trial. Diabetes Res Clin Pract. 2021;173:108701.)

Sincerely,

Tansy Edwards
